# Chaos in Western Medicine: How Issues of Social-Professional Status are Undermining Our Health: A Research Review on Issues of Medical Sociology related to Occupational Dominance of Medicine in Healthcare Environments

**DOI:** 10.5539/gjhs.v4n6p1

**Published:** 2012-07-25

**Authors:** N. W. Wilson

**Affiliations:** 1 Nathaniel W. Wilson, Medical Educationalist and Researcher, Cape Town, South Africa

**Keywords:** undergraduate medical education, medical profession, medical sociology, social-professional status, occupational dominance, medical and nursing hierarchies, medical qualifications framework, global health services

## Abstract

From the period immediately following the second world war, western (orthodox) medicine – both as a philosophy of medicine and as a professional guild of medical professionals actively practicing medicine – has made progress in leaps and bounds, especially considering the advances in technology and associated enterprises. Over the last thirty years, however, the practice of orthodox medicine has taken a turn for the worst despite progressive philosophies and tenets of basic practice as offered by the professional bodies that regulate how medicine is operated and implemented. Current healthcare environments are in a chaotic state of affairs, most notably due to issues involving affordability of medical professionals. It is argued that the social-professional status of medical doctors allow exorbitant and unreachable demands on governments for increased salaries. The title-based supremacy of doctors within the occupations domain is not supported by what they are offering society at large, and it compromises the ability of medical institutions and governments to provide better and more affordable healthcare. From a sociological point of view, this paper examines the social-religious history of such social class-based occupational power and dominance, and paves the way toward an overhaul of current medical education frameworks that proactively will ensure greater occupational equity in healthcare settings, across all healthcare disciplines tasked with patient care and improvement of healthcare services. In essence, doctoral titles should only be awarded after successful completion of postgraduate doctoral studies, and a new breed of medical professionals must emerge, able to contribute more meaningfully to the advancement of medicine as a profession, as well as toward increased standards of healthcare and improved health services delivery.

## 1. Introduction and Problem Statement

From the period immediately following the second world war, western (orthodox) medicine – both as a philosophy of medicine and as a professional guild of medical professionals actively practicing medicine – has made progress in leaps and bounds, especially considering the advances in technology and associated enterprises. Over the last thirty years, however, the practice of orthodox medicine has taken a turn for the worst in an already pessimistic global society, despite progressive philosophies and tenets of basic practice as offered by the professional bodies that regulate how medicine is operated and implemented. This mistaken turn, arguably, is responsible for the chaotic state of affairs we have in today’s healthcare environments across the globe. Current news headlines bemoan what has become of global healthcare, in the capacity to provide adequate services, to ensure increased levels of population health or to prevent an overall decline in population disease indicators. On one side of the coin a picturesque portrayal of healthcare is given, supported by statistics that indicate how new technologies are improving the quality of life through most advanced (and most expensive) medical interventions, but on the other, darker side, a more gloomy reality exists: more and more people are left aside with declining prospects of better health, associated with decreased levels of access to affordable and efficient healthcare services.

At the center of orthodox medicine stands the medical doctor (or general practitioner), and it is to this medical professional that we look as the ambassadorial provider of medicine and efficient medical services. Medicine – as a profession – is pre-eminent in the healthcare domain (Ham & Alberti, 2002), i.e. medical professionals are viewed superiorly compared to professionals from other healthcare disciplines. Also, as pointed out through a sociological lens into the world of professions, this pre-eminence extends over the general notion of professionalism, the world of professionals and professional occupations; in fact, medicine and law, as professions, have always enjoyed higher rewards, status and authority (Evetts, 2003). In analyses of professionalism it has been realized that higher (occupational) rewards were the result of occupational power rather than professionalism when focusing on medicine and law as the archetypal professions in Anglo-American analysis (Evetts, 2003: 400). The stark reality of our global healthcare situation is that ‘advances in medicine’ does not correlate positively with ‘decline in healthcare indicators’, in fact, the efficacy of orthodox medicine has been questioned for more than 40 years since Cochrane stressed the need for caution when evaluating the impact of medicine in the face of all the technological advances (Cochrane, 1972). When considering this conundrum it became apparent that deeper investigation into the sociological nature of medicine was needed to understand how our healthcare is declining amidst the so-called advances as advertised and promoted by the general healthcare industry.

Firstly, as with other occupations, medical doctors – supported and protected by professional guilds and unions – are generally considered by the public to be experts, or professionals, able to provide professional and efficient medical services (Crompton, 1990). However, the levels of occupational security for medical doctors are a few folds higher than for other occupations despite evidence that medical doctors and the medical world are not delivering professional services that are as effective and able to address most healthcare problems on a scale that warrant these high levels of occupational security. Secondly, it has been reasoned for many decades that the idea of professionalism (or the concept of professions) may be a weakened idea, a concept that is increasingly losing its power in the occupational world (Broadbent et al., 1997; Stichweh, 1997; Krause, 1996). Considering such a weakening of professionalism, it becomes a necessity to question that validity by which the medical profession is securing such a foothold within the global occupations domain, even amidst evidence that it is failing in its cause to establish itself as the authoritative voice in effective healthcare provision. Thirdly, and also the point at which we will focus our attentions during this exposition, the medical profession is the only such profession within human-oriented sciences where a prolonged period of undergraduate study is sufficient for the attainment of a ‘doctor’s title’, i.e. where the honorary titles “*Mr*.”, “*Mrs*.” or “*Ms*.” of the successful individuals are changed to “*Dr*.”. A change in title is a noble achievement (from both social and professional points of view), and immediately elevates the social-professional standing of the individual who attains it. Careful consideration of this matter allows one to posit that many individuals are studying medicine merely for achieving this change in professional title, and not for purposes, *per se*, of being a medical professional who can contribute meaningfully to the growth of the profession that could ultimately allow increased levels of effective service.

It is this poignant realization that indicates a pathway toward understanding many problems within the medical domain – at the root points of where these problems manifest themselves through the most subtle expressions as noticeable in situations of sociological interest. Herein specific reference is made to those problems that lie woven into both medical and social worlds, the kind of problems whose elucidation can explain why standards of medical service delivery are not at their best, as the professional medical authorities would want us to otherwisely believe by means of their renewed lip service and philosophies that call for increased healthcare service delivery and standards across the globe. These issues are highlighted by strong advocacy attempts, e.g. through millennium development goals (MDGs) or possibly the Framework Convention on Global Health (FCGH – cf. Friedman & Gostin, 2012). In essence, mere undergraduate study should not be enough to enforce such a change of professional title, as it predisposes the medical profession to be the ultimate home of ulterior-motivated professions-based exchange of services within the professional world: if one’s main aim is to achieve an expedited change of title, it should not be achieved through studying undergraduate medicine – the state costs per medical student are a matter of deep concern, especially if more than a third of all medical graduates never planned to practice as medical professionals (e.g. Burch et al., 2011). The state could use the funds in better ways if training of doctors would not be so costly, but more so if services of experienced, well-qualified medical professionals were more affordable. The spate of recent pay-related strikes and wage-centered public discussions indeed provide a firm support to the claim that the issues of status and money are of greater concern to most medical doctors than healthcare delivery itself (Blackwell, 2011). These service interruptions are chaotic, and leave many governments at a loss: most medical professionals are almost entirely unaffordable and most communities are left in dire need of basic medical services – services that are becoming increasingly out of reach of the majority of people in poorer and underserved areas.

As a result, orthodox medicine has been thrown into a state of chaos which lies at the hands of a deep underlying social issue involving occupation-driven class-based social hierarchies of individuals within different societies across the globe, affecting how healthcare services are managed and delivered. This chaotic state of affairs needs to be addressed, and this paper summarizes the initial thoughts on how to take the necessary steps in the right direction toward occupational equity across the wide landscapes of professions and professionalism, as well as toward increased levels of healthcare services across the globe. A brief overview of medical sociology is provided, followed by a problem statement and a more detailed exegesis that could provide an important stimulus-prompt toward a much-needed debate on a matter that deserves top priority within the ranks of high-level decision makers of the healthcare world.

### 1.1 The Issue of Professionalism and A Brief Overview of Medical Sociology

The definition of professions is a highly disputed matter, but Evetts (2003) provides a useful approach: “professions are essentially the knowledge-based category of occupations which usually follow a period of tertiary education and vocational training and experience”. Similarly, professionalism should be treated according to the very name or identity of the profession itself – in essence, professionalism would have a different meaning depending on which profession one is referring to. More importantly, the many different interpretations of professionalism are divided into one of two groups – as a normative value system or as a controlling ideology (Evetts, 2003). The normative view of professionalism is more optimistic about the contributions of a profession to normative social order, while the ideological view of professionalism focuses more negatively on what professionalism has achieved in the occupational and social domains by means of interpreting professionalism as a hegemonic belief system and a mechanism of social control for ‘professional’ workers (Evetts, 2003). Light (2000) highlights that already in the 1970s the cultural authority of the professions were shaken when a spate of books and articles appeared about the social pathologies of professional dominance, as well as neglect of the poor, of those with chronic conditions, and of public health issues (e.g. Kennedy, 1972; Ehrenreich & Ehrenreich, 1971; Greenberg, 1971; Nixon, 1971). In an eloquent manner, Light (2000) further describes the concept of a profession as a countervailing power in society, where the profession has a unique relationship with the state that is not necessarily beneficial to society at large, even if Jones (1991) already cautioned that such harnessing of professional work to the state’s goals can also lead to underfunding, perfunctory medical services, delays and bureaucratic hassles, demoralization, and the eventual deteriorization of medical practice, as happened in the prior decades, and as such is still happening today.

During the 1950s, Talcott Parsons – one of the main architects of the school of sociology known as functionalism – made a very influential analysis of the place of medicine in modern society (Parsons, 1951), so much so that most of our contemporary thinking in medicine can be seen as responses to his functionalist views (Lafferton, 2007/08). Through the eyes of well-known sociologists, the argument was put forth on how medicine has moved from ‘function to dysfunction’(Illich, 1995 & 1975; Mckeown, 1979; Zola, 1972) and that medicine’s social power and influence have long been understood to be closely related to its status as a profession (Abbott, 1988; Starr, 1982; Freidson, 1976). From the functionalist perspective, professions are regarded as institutions ‘that serve to ensure that their practitioners used their knowledge and skills to the greatest social benefit’ (Parsons, 1951), in other words, it could be said that the profession (of medicine) can be used to assure a greater social standing within the successful medical doctor’s sphere of movement. In more appropriate academic language, professions are viewed by sociologists as institutions for securing occupational and social authority, and dominance (Freidson, 1986; Starr, 1982). Ultimately, and unfortunately, the state of contemporary orthodox medicine, as well as the chaotic nature of its social business and practice, can succinctly be captured in industrialized terms such as bureaucracy, organization of mass medicine, medicalizing of society, the sick role, medical consumerism, and medical social movements (Lafferton, 2007/08). The overwhelming cause of concern is this dominance of the medical profession both within the wider domain of general healthcare and outside of it – perpetually imposing the perceived authority and dominance despite growing evidence of its ultimate failure to provide increasing standards of service and subsequent increased states of health across the globe. Still, medicine (or medical professionalism) continues to be regarded as pre-eminent amongst the healthcare professions (Ham & Alberta, 2002), and most often excludes the nursing, pharmaceutical and allied healthcare professions even if strong interrelations and cooperation between these professions are of central importance toward achieving more progressive health outcomes.

Thus, as a historical phenomenon containing a high degree of socioeconomic complexities, the status of medical professionals within the healthcare domain has been a major stronghold on policies and tenets of medical practice, simultaneously disguising itself as an innocent criterion of medical professionalism while maintaining a secure vice and damaging effect on the practice of healthcare as a whole. A survey of the current purview of medical literature, including medical sociology, seems to suggest that the exact nature of the title-based elevated status of medical professionals is overlooked in discussions regarding the effects of professionalism on practice, hence the reference to it here as the inconspicuous monster lurking beneath the once-glossy surface of medical practice. It is to this end that the issue of medical professionals’ status becomes a major undertaking in the ensuing discussion: in order to rid medical practice of a most destructive social vice, we need to study and understand the precise nature of the unjustified elevated status of medical professions, and with this endeavor must emerge a new understanding of issues pertaining to medical professionalism that impact on our daily lives.

### 1.2 Problem Statement

When students graduate from secondary schools, they are confronted with a plethora of choices. Unfortunately, most students never consider the whole range of possibilities across the adult work domain and, most frequently due to pressures within families and societies, consequently dive into the *highest* tiers of what is commonly referred to as *top notch* professions, e.g. medicine, engineering, law, accounting, architecture and actuarial sciences – meaning that most students would only be considered successful if they, for example, become doctors, engineers, lawyers, accountants, architects and actuaries. What most people fail to realize, or fail to admit, is that a bank clerk could also become a bank manager, and that a 2-year diploma is often a sufficient investment, initially, to enter the banking industry, as a means to become a highly successful bank manager, providing the candidate is sufficiently strong (emotionally and cognitively) to survive the competitiveness within the banking industry. In the end, the bank manager, with a lower academic qualification, will employ a more highly qualified accountant. Somehow, the entitlement to luxurious lifestyles and wealthy dispositions have developed as a main outcome associated with the achievement of professional titles, and this entitlement, or the different degrees of entitlements, as it stands to reason, is the factor responsible for the majority of inter- and intra-professional lines of distinction that run between and through different professions^1^.

From a human healthcare^2^ point of view, most people generally consider you successful only if you manage to achieve the MBBS (MBChB) or BChD degrees, which then *transform* you from a normal, educated citizen into a medical doctor or dentist. A natural evolution in the social status of the successful medical doctor is immediately apparent – the “*Mr*.”, “*Ms*.” or “*Mrs*.” now becomes “*Dr*.” – the “medical doctor title” has now evolved to be the ultimate status symbol within the healthcare (or medical) environments, and in itself becomes a primary target of achievement. The former fact (achieving a medical degree) is not the problem; it is the latter situation that causes most distress: the situation that arises from enrollment of most medical students^3^ into medical programs in order to become “doctors” with “doctor titles”, whilst knowing that they were never planning to practice as medical doctors beyond successful graduation (Burch et al., 2011). A more prevalent basic assumption is that the doctor title entitles the successful candidate to a more competitive salary, regardless of the field of endeavor that follows the medical program. In reality, and perhaps forgotten over recent years, the MBBS is the entrance requirement into postgraduate clinical medicine, but a grieving knowledge exists in the fact that a small percentage of qualified medical doctors actually participate in the capacity to contribute more directly to the advancement of medicine, as a whole, also considering the negative social impact of a declining effectiveness of medical personnel and institutions. Interestingly, when interviewed on issues concerning job satisfaction, income is regarded as one of the most prominent features amongst medical personnel (Goetz et al., 2011; Wilson et al., 2009).

Thus, our main problem, for the purposes of this treatise, is the doctor’s title awarded to medical doctors, after only six years of undergraduate training, combined with title-based pre-eminence in both the healthcare and general job markets, and the over-emphasis on income when asked about healthcare provision and job satisfaction, which renders them (as public servants) affordable only at unacceptably high salaries. To this problem we need a solution, for the sake of an improvement in the general state of population health across the globe. The status of population health must enjoy top priority status; it is thus time for the medical profession to do justice by the public in the way it does training of its professionals – through altering and improving the mechanism by which it awards the sought-after qualifications – all to ensure a more rigorous professional accountability that adapts to the changing world, now, in 2012, which is different from the time immediately succeeding the second world war. Ham and Alberti (2002) called for a reform of the NHS, whereas this review calls for an overhaul of the healthcare qualifications framework as it currently stands, with specific focus on the medical qualifications framework, as well as for an even more serious effort toward an integrative global healthcare qualifications framework.

## 2. A Historical Context on the Role of a Physician and the Development of Social Hierarchy Systems in Healthcare Environments

Before addressing the changes that are needed in the medical qualifications framework (Section 3), it is necessary to highlight a few issues that are relevant in a more sociological context of medicine, namely the issues of social class and subsequent class-based social divisions amongst people in spheres of human activity. In terms of proving one’s claims about class-based separation and division, it is difficult to find reliable evidence to support a theory that medical doctors are ‘raised’ or ‘elevated’ from status of normal civilians to public servants of the highest regard solely by virtue of the academic qualifications they hold, yet almost every culture has indeed provided the necessary platform for doctors to be regarded as ‘professionals of the highest skills and caliber’ (author’s own caption) – which in turn only enhances the social profile of the medical professional even in the face of failure to provide a competent and reliable medical service. Admittedly, finding such evidence will most probably be regarded tantamount to provocation of the highest order by medical professional bodies, and it may undermine much of the progress made over the last few decades, if one is indeed able to see significant progress in any specific area of medicine under discussion. Yes, there have been advances in technology, and yes, we have increased the level of technical knowledge regarding the human body, and yes, there have been major improvements in understanding the mechanisms of many medicines and natural cures to ailments and disease, but – despite all these *improvements* – we face a dire situation in which outcry after outcry has been sounded, calling for more efficient and improved medical services, and especially for more affordable healthcare. These outcries against what the medical profession is doing – as set against what we expect them to do – are becoming louder and louder, and has been gaining momentum since Parsons made his analysis public. The latest such attempt is shown in the work of Freidson and Gostin (2012), and it shows very serious attempts to incorporate the *right to health* into national law and policy. The perceived notion of the FCGH may indeed underscore many of the cries made in the circles of renowned sociologists, and its eventual success could overturn the nagging inequalities with which healthcare is currently plagued.

In an attempt to understand the sociological nature of the elevation of medical professionals’ status, it was necessary to weigh the *improvements in medicine* (as claimed by the profession at large) against the *outcries concerning the unacceptably low levels of efficient healthcare services* as evident by the increasing burden of disease and poor epidemiological statistics. The important question was, ‘How could a profession, claiming improvement and advances in most of its qualifications, skill sets and procedures, manage to lead the healthcare world into a state of despair, possibly bringing the state of orthodox medicine – and its appeal – to an all-time low’? After some contemplation the most obvious answer came in the realization that perhaps there is no direct link between the publicized advances in medicine and the unacceptably poor states of health that exists in communities throughout the world. It could well be argued that technological advances in medicine mainly exist for other purposes than for improving medical services, such as allowing the medical profession to be more profitable – an idea that has already been proposed and accepted amongst well-known medical sociologists supporting a Marxist theory of capitalism (for a detailed analysis see Navarro 1985, 1977, and 1976). In fact, improvement of medical service efficiency and standards could be a low-priority prospect amongst the medical professionals within most institutions, a realization which in itself causes grave concern about the future of orthodox medical care. Perhaps there is an indirect link, a hidden factor – even a missing link from outside the normal confines of medical practice – that could aid our understanding of the medical profession and its role in bringing our once-thriving healthcare systems to its knees. It was in search of this missing link that some thoughts were spared to the origins of the physician’s role in mankind’s quest for improved health, and after considerable pondering it became clear that a deeper social-religious basis exist for the elevated status of doctors, which is discussed in the section below.

### 2.1 “The Great Physician Is Nearby…” The Role of Religion and Beliefs in Forming Expectations of Professional Superiority

As is the case with most social problems, our problem has an extensive history that spans a lengthy period of time, specifically since biblical times. The title of this paragraph hails from interpretations inferred from texts in the Christian Holy Scriptures (cf *The Bible*), made in reference to the Jesus Christ, as was engrained into the hearts of millions of Christians and religious people across the world, and as studied by those interested in the effects of religion on society at large. Jesus Christ often was known for referring to himself as a physician, to heal the sick, as evident by the verses as written in Luke 4:23,

“…and he said unto them, ye will surely say unto me this proverb, Physician, heal thyself: whatsoever we have heard done in Capernaum, do also here in thy country.”

A similar self-referral is found in the gospel according to Mark 2:17,

“Upon hearing this Jesus said to them: “Those who are strong do not need a physician, but those who are ill do. I came to call, not righteous people, but sinners”.”

In fact, in the same manner that people during biblical times are believed to have held high expectations of omnipotence from Jesus with regards to the ‘healing of ailments’ (physical or spiritual), contemporary physicians are often expected to ‘know-it-all’, displaying that same kind of omnipotence when it comes to informing on prognoses and diagnoses, owing mainly to the mere fact that medical interventions, and the successes thereof, could translate into the difference between staying alive for a longer time; or dying – if the medical intervention is not successful. It is often believed, from a background of religion, that a western doctor is a most important part of your life, as important as your religion – also backed by state accreditation, which sets the orthodox doctor apart from any of the other healthcare or medicinal experts. When a doctor does not know how to explain a certain medical ailment, and when the prescribed medicine does not show to work (or worse, it worsens the prognosis), the only other solution is to turn to the Higher Being. This automatically *elevates* the state-accredited western-trained medical doctor to the place directly below the Higher Being, as a *demigod*. In stark contrast to this, a new awareness level of CAM (complementary and alternative medicines) has been reached in progressive circles, but not enough to offset the reliance on orthodox medicine, where doctors “know better than anyone else” within the medical setting, or worse, they “claim to know better”. As an added occupational hazard, the average westernized doctor often abates and denounces any of the other, possibly more effective approaches to healthcare. There are, however, well-informed orthodox medical doctors who have accepted CAM and its applications, in principle, some of whom who would allow their patients to enjoy the best of both approaches (Joos, Musselmann, Szecsenyi, & Goetz, 2011). Thousands of millions of followers of the orthodox doctrine still will not accept a herbal concoction that may benefit them, because “*my doctor said it’s bad*”; they’d rather ingest a synthetic chemical supplement that could cause further destruction within their bodily systems – because “*my doctor said I should*”. Interestingly, CAM practitioners are often considered to be witchcraft doctors, especially in more primitive and traditional settings, relying on some kind of magic (*vs*. knowledge) to arrive at certain conclusions concerned with prognoses or diagnoses. To make matters worse, medical doctors, with limitations of knowledge, are constantly set against the expectation that all answers are readily available, and that any non-achievement of an immediate demonstration of accurate knowledge is subsequently equated with incompetence. As a result, medical doctors, at an increasing prevalence, face themselves in courts and other instances of legal persecution, leaving trails of paperwork and a disgruntled public. In essence, from a global point of view, the morale surrounding effectiveness of western medicine is low, possibly at its lowest point in human medical history. By our collective understanding of these issues we could take ourselves forward toward better health through enhanced global cooperation that will ultimately rid our healthcare environments of an unnecessary vice.

### 2.2 Medical Hierarchies and Its Prominent Placement in Healthcare Settings

**Diagram 1:**
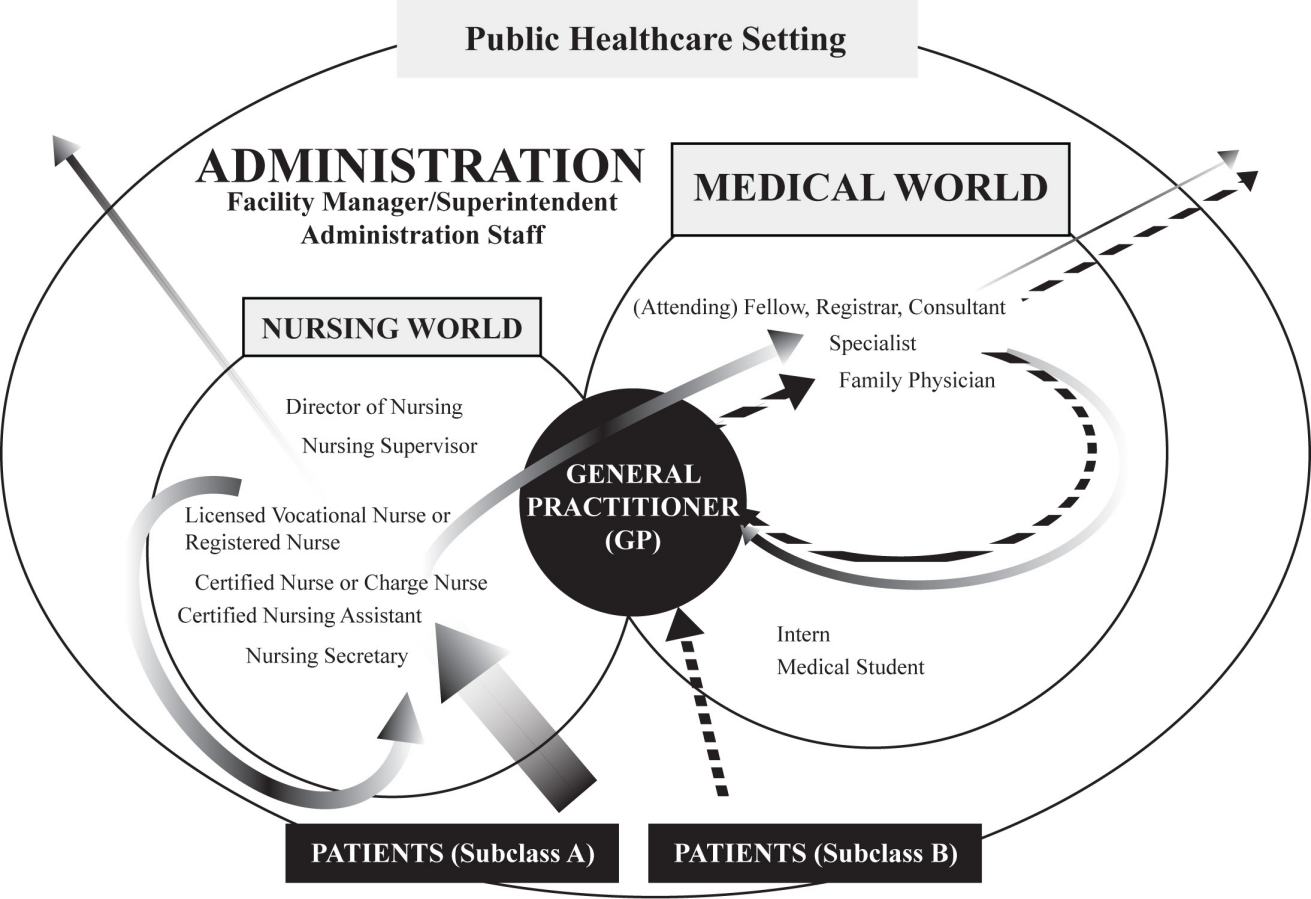
Medical and Nursing Hierarchies in Typical Westernized Healthcare Settings. Medical and nursing worlds are depicted, implying class-based occupational hierarchies that determine (i) how patients from two distinct subclasses flux through a medical setting, and (ii) the status-based occupational division of workload and responsibilities within the healthcare setting. Arrow thickness represents estimated percentages of the two patient subclasses relative to total patient population as seen across the scope of operations within a typical healthcare setting. For purposes of discussion, the relations to allied health and paramedical occupations are not depicted.

The previous section briefly outlined a historical development in societies that could explain the perceived superiority that medical professionals continue to enjoy in both social and occupational settings. Coupled to this issue of occupational superiority is the issue of titled-based hierarchies in the workplace. The title of this paragraph refers to the hierarchy as encountered in the typical *world of medical doctors*, hence referred to as the *medical hierarchy*. Alongside this medical hierarchy exists a parallel hierarchy in the *world of nurses*, too, which is not the focus of this paper, but is included to serve as a context for comparison. The latter hierarchy can be referred to as the *nursing hierarchy*, and is strictly adhered to even within the confines of a general healthcare facility, where doctors and nurses are found within the same setting, the doctors not always being the leaders and nurses not always the followers, a situation that most would accept as improper. Based on experience, these two hierarchies are, at most, mutually exclusive. Although our problem focuses on the medical hierarchy, it must be remembered that, in reality, the effectiveness of medical services offered by doctors are always dependent on the dynamics within their pool of colleagues consisting of nurses and other allied health professionals, but mostly the nurses. It suffices to say that the strict hierarchical nature of positions and titles, within the general healthcare setting, does provide many challenges that impact on the level of medical and general healthcare services that the facility can offer. Diagram 1 positions the GP in the center of the medical world as well as in the centre of the public healthcare setting, and Table 1 contains information about hierarchies, collected from social websites, underscoring the claim that these hierarchies affect how medical professionals deliver their services. The medical and nursing worlds are each assumed to have distinctive outcomes that may not necessarily be tied toward the same healthcare outcomes. On the opposite side of this distinction lies the concept of an integrated healthcare setting, where both medical and nursing worlds work interdependently toward the same outcomes for patient care. Of course, from academic or economic points of view, the existence of hierarchies is not the main problem, or it could not be a problem at all. Greater academic and clinical skills should be accompanied by a higher salary and the appropriate recognition by the employer, the public and the professional authorities. However, the problem of social-professional status-based hierarchies is multi-faceted if viewed through a sociological lens.

**Table 1:** Medical and nursing hierarchies as summarized on blogs and social websites

Source	*ehow.com*	*medstudentsonline.com.au*	*medschoolhell.com*	*allnurses.com*
Top of hierarchy	hospital administrator	hospital executive	attending	director of nursing
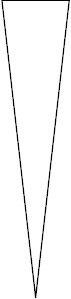	specialist surgeon	heads of department	fellow	nursing supervisor
	normal specialist	consultant	resident	licensed vocational nurse, registered nurse
	silent doctor	senior registrar/fellow	intern	charge nurse or certified nurse
	nurse	advanced trainee	nurse	certified nursing assistant
	(physician assistant, GP)	non-advanced trainee registrar	medical student	(unit) secretary
	medical student	resident medical officer		
		intern		
	comment:	comment:	comments:	comments:
	not sure where to place physician assistants	**quote** “nurses have their own [hierarchy]; the senior clinical nurses are often more experienced, seen when the consultants were residents and know a LOT more in their field than you think you do”	equates medical school hierarchy with “totem pole”; does take “nurse” into account	not sure whether licensed vocational nurse enjoys higher status than registered nurse
Bottom of hierarchy				**quote** “technically speaking, hospitals aren’t hierarchies” – see **Appendix 1**

Data collected from social websites and blogs concerning hierarchical structures in medical and nursing worlds, with comments to illustrate personal viewpoints or comparisons relevant for discussion as offered in the main body of text

Firstly, and immediately apparent from Diagram 1, is the distinctive areas of demarcation between the doctors (in the medical world) and their “followers”, namely the patients, nurses (in the nursing world) and professionals from other health professions (not shown in diagram). There is a thick invisible line of separation between these different *worlds*. This line is an important line; it has many shades of hidden meanings and is used primarily to categorize people into different social classes notwithstanding that in most medical environments, it is the nurses who assist in orientation-related training of doctors. Nurses are, then, for the purpose of this paper, the most undervalued healthcare professionals. From this point of view, general nurses, in hospitals, are treated in similar ways as teachers are treated in schools and daycare facilities by respective administration authorities and other public stakeholders. The first part of our problem with status, therefore, has something to do with classifying each other into social classes of individuals that may or may not be deemed appropriate to work alongside each other. This transforms our issue of titles into a similar problem that the famous Pierre Bordieu and Michel Foucalt are known for addressing, as summarized and eloquently clarified by Stephen Ball (2006) – the issue of class theory and class analysis with emphasis on the interactions of space and social networks and class formation and reproduction. As in Appendix 1 (Comment 2), it is almost as if “nurses are middle class citizens, doctors are upper class citizens, certified nursing assistants (CNAs) are lower class citizens, and the administration is the government in hospital land… etc.” The webpages from the website links in Table 1 can be consulted to appreciate the negative sentiments expressed in association with this issue. It remains to be seen by what exactness of degree these negative sentiments abound.

Secondly, and perhaps more disturbing, is the conceptual reality that people from all over the world are competing, at very high levels of competition, to gain access to medical training, only to the end (for an uncomfortably significant percentage of enrollments) of obtaining the change of title. With cultural capital as an accepted social construct it is easily conceivable that families with an already wealthy cultural disposition (most often accompanied by greater economic resources) are enabled to perpetuate their cultural and economic dominance in respective societies. The change of title could be an expedited means toward achieving a change in social class, and if studying medicine is the expedited way toward changing your social class, then the medical profession should put an end to this destructive social trend. The matter is severely exacerbated with the added conceptualization that medical graduates, in most cases, as proven by some preliminary research, do not intend to use their difficult and expensive training toward actual improvement of medical sciences and general population healthcare in areas that in dire need of those skills and expertise (Burch et al., 2011). If this were the case – if medical graduates were actually interested in the advancement of healthcare, as medical professionals – then the general healthcare world, at current, would not be inundated by such a large number of strategies and huge government expenditures with regards to issues such as workforce retention, medical brain drain and global migration of much-needed healthcare professionals, especially in rural and underserved areas (Wilson et al., 2009). After all, from a traditional point of view, choosing medicine was usually equated to fulfilling a life’s ambition alongside a very narrow list of serious professions. When one surveys current medical literature, one does not gain the same level of appreciation for the level of work done by contemporary medical doctors because it is much easier to find pleas that call for change based on dissatisfaction with medical services or the affordability thereof. The ladder in medicine can stay the same, but the wall must be changed against which it is hoisted. In essence, healthcare professionals should walk the same road to a doctorate as doctoral candidates in other major academic fields of endeavor, if the primary outcome is to change your title. Achievement of a doctoral degree is accompanied by a major contribution to the candidate’s chosen field of study, and only candidates who show the ability to defend a doctoral thesis should be conferred with such a noble change of title.

Thirdly, the medical and nursing hierarchies have spilled over into the patients’ sphere of reference, so much so that it has created a subclass system of hierarchies. When you make the time to engage patients in a casual conversation about healthcare, they will accurately explain the chain of command amongst the medical professionals in the setting. Personal experience allowed the impression that patients, whether consciously or subconsciously, perpetuate this class-based separation by classifying themselves according to the level of medical care they are deemed appropriate to receive. The patients, too, divide themselves into different classes, emulating the classes of those medical professionals under whose care they have been placed. This is evident by the many patients who will tell you that “*my doctor* [emphasis] is a specialist, and *he* is the only such specialist in this whole region, and *he* said I’ll be well if I just take my medication every day. My daughter is a nurse and she tells me the medication is not helping in any way, but she is not the doctor – *my doctor* says I must just continue taking the medication. I listen to *my* doctor”. Unpublished research-based experience allows one to conclude that this type of class-based association happens especially amongst patients from subclass A (Table 1), from their assumed lower economic status and associated lack of ability to choose a higher level of care. Patients from subclass B might have an improved understanding of their level of choice and the distinction between different levels of medical care, even if only slightly. Even more telling is the physical structures of the hospitals or clinics, where the architectural designs of the buildings have been used to divide the sections in such a way as to keep the medical and nursing worlds separated from each other (Gillespie, 2002). One of the possible spin-offs from such a system is that patients from subclass B (or, better, their medical insurance companies) will pay much more for medical services in the medical world even if the same services are available in the nursing world at lowered premiums; this can mainly be understood if one assumes that there exists a strong class-based separation between patients, or between the medical professionals providing the service, and that people consider this type of distinction to be very important, perhaps even more important than the issue of exorbitant amounts of money that insurance companies will pay on behalf of patients for basic services.

Ultimately, the social class-based categorization swallows even the higher levels of decision makers, where governments and local authorities are, for example, spending huge financial and human resources aiding toward certain predetermined outcomes, such as making sure that a fixed percentage of subclass A patients are catered for by public facilities, and that other public-private service providers are allowed to cater for subclass B patients, or the occasional patient from subclass A who can afford the additional premiums. It is a vicious cycle of class-based discrimination that is currently undermining most of the more progressive attempts at improving the levels of healthcare provision. Strikingly, from the point of view of affordability, one can assume it is virtually impossible for any government to afford all the the doctors they need if one uses the most recent information indicating the salaries of doctors: calculated at between $100 000 (US) and $450 000 per year (Blackwell, 2011), a medical career spanning thirty years can cost the government between $3 million and $13.5 million per doctor. This excludes funds for undergraduate training. It is not clear whether these statistics include additional monetary benefits, but after making the calculations based on these figures a very clear picture is painted with regards to our biggest problem in contemporary medical care. Is it too difficult to conceive that a skewed focus on achieving better social stature and monetary wealth by way of a professional job title (on the part of the medical professional) can ultimately be responsible for a wide array of problems that cripples the very system that the medical profession professes to want to improve? In fact, the entire healthcare setting, as outlined in Diagram 1, is set up to support the medical world in achieving its undue prominence in the healthcare world, leaving in its wake an ailing population served by inadequate human resources and alarmingly poor levels of professional service.

## 3. Reshaping the Medical Qualifications Framework

In an attempt to address the problem of occupations-based status, as outlined above, it was necessary to take a step back and consider current orthodox medical qualifications frameworks, all mainly drawing from the Maastricht model of medicine (Bouhuijs, 1978) and adapted for each continent, country or medical school since those years of medical school reform. Based on most recently available information on curriculum frameworks, it seems that one or a combination of a handful of models are most widely adopted and implemented when offering undergraduate medical programs: Germany, United Kingdom, United States of America, The Netherlands and Canada (Chenot, 2009). Contemporary reform of medical education has been suggested, and early steps toward long-term implementation has been taken (Hays, 2007), but specific details of undergraduate medical programs are found with great difficulty. In the same manner that elevated status of medical professionals are not directly addressed in current literature, it also seems to follow that any details pertaining to the overarching qualifications frameworks are widely elusive. In the dentistry profession an exemplary step toward common frameworks is provided by Sanz, Widström and Eaton (2008), calling for increased collaboration between competent authorities, including governments, universities, dental associations and the various Pan–European Scientific Specialist Organizations. As Karle (2006) states, the World Federation of Medical Education (WFME) acknowledges that

*“globalization of medicine is increasing, as manifested by the growing number of migrating doctors and cross-border education providers. In addition, new medical schools of dubious quality are proliferating. This situation accentuates the need to define standards and introduce effective and transparent accreditation systems”*.

However, since this admission, relatively little has been done or implemented to show the way forward. It is further claimed that “information about accreditation status-agencies involved and criteria and procedures used will be essential to future databases of medical schools and [as such] will be a foundation for international meta-recognition of institutions and programs (accrediting the accreditors)” (Karle, 2006), but sadly this has not changed much over the last six years. MacCarrick, Kelly and Conroy (2010) reports an institutional self-review using the WFME standards although this single report poignantly highlights the great lack of reported effort throughout the rest of the world. There seems to be a severe inertia in academic medical education circles that prevent suitable action toward attainment of these desirable outcomes. Even if this inertia is caused by socioeconomic or political influences, more needs to be done, globally, to break the wave of procrastination that currently inhibits our growth toward more effective solutions.

**Diagram 2:**
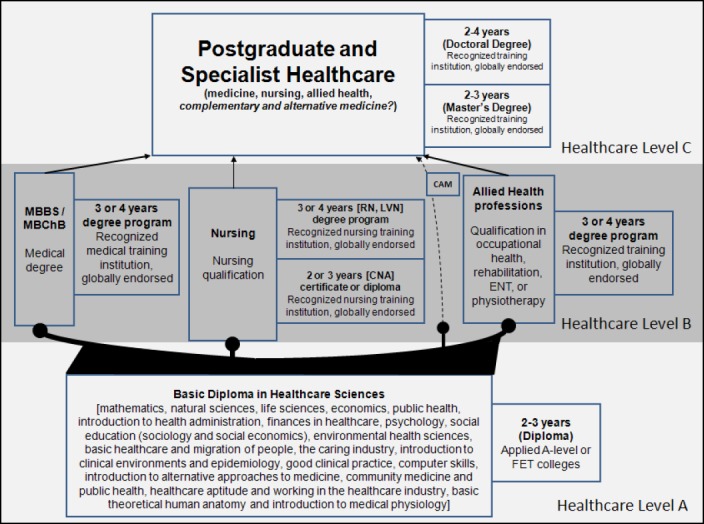
An Integrated Holistic Healthcare Educational Framework. The diagram presents a renewed conceptualization of a globally recognized healthcare educational framework, dividing the overall healthcare training platform into three distinct tiers, viz. Healthcare Levels A through C, as discussed in the main body of text (Section 3). Not shown in detail is a separate tract for specialization in complementary and alternative medicines (CAM) that can possibly be added to Healthcare Level B (dotted curve). More importantly, within this new dispensation of healthcare qualifications, the Basic Diploma in Healthcare Sciences is suggested as a mandatory requirement for certification in all Healthcare Levels B and C programs; in addition, doctoral titles are to be awarded only after completion of postgraduate work by means of a doctoral thesis or equivalent mechanisms – as currently required by researchers from other academic or occupational domains. Abbreviations: CNA: certified nursing assistant; ENT: ear, nose and throat; FET: further education and training; LVN: licensed vocational nurse; RN: registered nurse

It seems, consequently, that a gap of action has been left in the current world of academic medical training and accreditation – if such a gap is allowed to grow, it will surely continue to exacerbate the crippling of our healthcare systems amidst the ever-growing demand for more affordable and effective healthcare services. As a result, a forced consideration of qualifications frameworks in one’s own medical environment is due, with comparison to other medical qualifications frameworks across the globe – using information that is available through university and government websites – a very laborious effort indeed. Taking into consideration the issues of social class-based hierarchy in medical settings, it was imminent to form a hypothesis that no course correction would be possible if we continue to separate the title of doctors from issues of medical curriculum-based accreditation, also seen from the point of view that the medical profession is the only such profession within human sciences to award a doctor’s title after completion of undergraduate studies. After surveying different curriculum frameworks, which do not differ so much in content as they differ in structure, a novel conceptualization of a healthcare qualifications framework became apparent. With no specific published model as a direct guide, but using the information as published on medical school websites across the globe – representing current medical, nursing and allied health professions’ qualifications frameworks across different continents – Diagram 2 shows a scheme derived from a combination of such information and the quest for effective change, for use as guide in initial steps towards reshaping the current mode of healthcare qualifications frameworks. The main difference, conceptually, with previous medical qualifications frameworks is mainly the insistence on postgraduate training and PhD-level certification as the minimum requirement for achieving a doctoral title. Subsequently, one of the main issues will be of a more practical nature: will medical professionals with a four-year undergraduate degree receive similar levels of income and recognition as professionals in nursing and the allied health professions with the same amount of training and accreditation as, for example, registered nurses and senior occupational therapists who studied respective four-year programs? A four-year medical degree allows entrance into postgraduate medicine, and only after successful defense of a doctoral thesis will the appropriate doctor’s title be awarded.

In this new characterization of a more integrated and holistic healthcare education qualifications framework, healthcare education is divided into three levels – Healthcare Level A, a basic secondary-level education comprising a more holistic training approach aimed at developing the type of individual who could understand the wider implications within the career field of choice; Healthcare Level B, a specific tertiary-level education (university or post-secondary higher education in some countries) comprising a more specific professions-driven focus, directly building on the core foundation as laid in Healthcare Level A; Healthcare Level C, a postgraduate-level education aimed at building research-minded individuals who will contribute meaningfully to the development of their disciplines and professions within the wider healthcare domain. Healthcare Level A resources will mainly consist of theoretical approaches since its implementation would be aimed at secondary education environments, whereas healthcare levels B and C will maintain similar physical infrastructure resources as currently enjoyed, merely as a means to limit the disturbances that an altered qualifications framework can cause to existing structures. Perhaps worth consideration is a separate qualifications tract in Healthcare Level B, for globalized state-accredited healthcare professionalization within complementary and alternative medicines, shown in the diagram as a dotted curve. Inclusion of a CAM tract in the official healthcare accreditation scheme would steer significantly toward the concept of integrated healthcare solution. Nevertheless, the scheme was designed to show that without Healthcare Level A-education the whole system of healthcare training would be inadequate, as without such a proper base of training we could run the risk of perpetually producing individuals ill-prepared for the life-long career-oriented tasks at hand. Before delving into the specialization of choice, no healthcare professional can be considered adequately trained without a solid socio-academic basis contained within the proposed basic, secondary-level education. Healthcare professionals work with people, in social situations, offering a science-based healthcare service involving many different levels of healthcare solutions ranging from primary healthcare to the highest levels of clinical interventions; these influence the lives of patients in an infinite number of ways – the most important of which has to do with quality of life and longevity. We need to proactively ensure that the healthcare professionals of tomorrow are better suited and prepared for this huge undertaking.

## 4. Moving beyond 2012: The Way Forward to Global Health

Table 2 not only shows preliminary goal setting goals (or objectives) toward overhaul of healthcare qualifications frameworks, but also provides possible strategies that could aid the timely development of this necessary process that could contribute significantly toward improvement of healthcare curriculum-based global service standards, as well as toward improvement of the quality of healthcare provision by qualified healthcare professionals. We must take into account an equalization of professional status of medical doctors compared to other professions. Of particular concern is the integrative approach that combines a suitable depth of knowledge, on the part of novice medical professional, with regards to mainstream orthodox approaches to medicine and the plethora of alternative approaches from across the world. Healthcare provision is increasingly becoming an integrative problem, and integrative solutions are the most reliable, immediate avenue of approach in our efforts to address current failing healthcare systems.

**Table 2:** Objectives and strategies toward medical and healthcare qualifications frameworks

Objective or goal	Strategies toward initial implementation
Initiate debates regarding medical qualifications framework	Invite all major role players within healthcare to participate in idea-sharing conferences and workshops; collect idea assessments and data to inform due process [WFME, FCGH, governments, university authorities, medical school leadership, medical unions and agencies]

Choose a healthcare qualifications authority to oversee the qualifications reshaping process	Healthcare leadership to engage and decide whether current authorities are adequately enabled to take responsibility for an overhaul in the medical/healthcare qualifications frameworks; if not, brainstorming could lead to establishment of a more unified global healthcare qualifications framework authority

Gain worldwide collaboration	Invite governments and healthcare controlling bodies across the world to agree and participate in reshaping the healthcare qualifications landscape; also to ascertain the time period needed to incorporate a new globally-regulated, and globally-endorsed qualifications framework into existing structures [this could aid in establishing more agreement between healthcare authorities across borders; could possibly reduce the long-term expenditure on certification of foreign-qualified healthcare professionals]

Configure a timeline for implementation of new qualification structures	Setting a date for when the enforcements of new qualifications requirements will be effective; also deciding on the number and level of pilot projects at leading institutions, to inform due process

Ultimately, this paper critically seeks to inform on the necessity of an overhaul of current medical and healthcare qualifications frameworks, in the attempt to improve healthcare delivery standards and the quality of medical care in the face of failing healthcare systems across a wide range of territories across the globe. At the center of this chaotic state of affairs lays, arguably, the issue of how social status and class-based categorization of people and healthcare professionals are hampering progress and development across the varied healthcare landscapes. The general medical doctor has been, for a very long time in the history of mankind, superiorly placed within the world of professions and has, in its wake of title-based supremacy, contributed to many low-performing outcomes within healthcare settings by a skewed focus on matters of social importance rather than the more pressing issue of healthcare services delivery of the highest standards – even despite taking the Hippocratic Oath, an oath that promises a high ethical responsibility toward the improvement of health and the well-being of one’s fellow man.

The practice of medicine is based on a scientific approach yet the outcomes of medicine and healthcare, in general, lay comfortably within the realms of religion, sociobiology, economy and psychology; it is these cross-links between socioeconomic, religious and scientific approaches that make the accomplishments of healthcare initiatives very complex. If the medical profession were to achieve what it set out since the end of the Second World War, this call to an overhaul of its qualifications frameworks would have been unjustified. Yet, it is exactly the non-ability of medical institutions and professionals to raise the current, appalling standard of global health and related issues that necessitated this call to arms – current interventions and attempts to save face are not convincing; it seems the social aspect of medicine and status of medical doctors have eluded the western world, although the impact of this dark, inconspicuous monstrosity has been living with many unsatisfied patients for years, some of whom sadly paid an even higher price for trusting their chosen healthcare professionals to assist in improving the general quality of life. It is time that the bare issues – not cloaked in empty, superfluous language of typical academic discourse – are put forth in a manner that will necessitate a global critical mass of movement toward improving an ever-failing system of health underpinned by false senses of entitlement and social superiority.

An overview of hierarchy systems within medical and nursing worlds is presented, drawing attention to a very serious cause of underlying tension and frustration that typically characterizes the westernized healthcare establishment. If conceived that such high levels of tension can abound while healthcare professionals are expected to deliver decent standards of care, then it is only logical that the cause for such tensions must be removed. It is currently impossible to accurately gauge the exact nature of this title-based tension – humans have shown an inherent incapability in sharing concerns about such personally challenging social issues in a trustworthy manner – and the presumption must be made that it is better to solve this issue in a more direct, proactive way rather than trying to address it after so many years have elapsed while the dark side of innate social competitiveness has wormed its way into the very fiber of our subconscious collective thought. This paper seeks not to destroy the positive efforts made by smaller groups of healthcare professionals within an even smaller number of existing healthcare settings – for which they are not always commended or acknowledged – it seeks to address the overwhelmingly negative attitudes and persistent resistance against positive changes toward a significant improvement of global healthcare status. Unfortunately the social wounds have abscessed too much for us to still only address the issue using our best words or other non-effective punitive measures; it is only by proactive means, in the form of a necessitated overhaul of current qualifications frameworks, that we could possibly move one necessary step closer toward a better state of healthcare for all.

Are we going back to Parson’s (1939) emphasis on institutional context, implying that “if we want healthcare that we can trust when we are sick and vulnerable, the body politic has to help the profession be as trustworthy as it would like to be, but cannot be on its own”? A new breed of medical officers are needed, those who are committed to committing a sizeable period within their career paths toward the practice of healthcare, and toward the advancement thereof. Of course our problem is not easily isolated from many other issues with similar symptoms as the one described above, but in essence we should make every attempt at rooting out the problems that are causing our familial and societal healthcare systems to fail in terms of its inadequacy to address our healthcare needs, or its persistence to continually place us at an arm’s length away from the most appropriate levels of healthcare that should otherwise be within our grasp. In answering the question “What shall we do?” (Light, 2000: 212), and within the confines of my vocation as medical education researcher, this paper calls for an overhaul of the healthcare qualifications framework as it currently stands, with specific focus on the medical qualifications framework, in the same frame of mind as Ham and Alberti (2002),

“*In an era of team working, medicine can no longer stand above and on one side from the collective responsibility to deliver high standards of care, even though the role of medicine among the health professions remains pre­eminent. Many do not wish to—but for others it is time to stop grieving for the past and to meet the challenges of the new world and the future. To be sure, the difficulty for the medical profession in acting in a concerted way in this debate is formidable, given the wide range of bodies like the BMA and the royal colleges that speak for doctors, but the risk in not doing so is even greater”*.

## Footnotes

1 For a list of professions see http://en.wikipedia.org/wiki/Profession

2 Here, as with the remainder of the paper, “healthcare” is used to include medical and clinical care, as medicine is considered pre-eminent within healthcare; healthcare is more generalized and includes medicine, nursing and allied health professions; healthcare thus including all aspects of physical and mental health, from basic socioeconomic-driven primary care to the highest levels of tertiary medical interventions. Some see medical health as top priority while other consider general healthcare a more holistic enterprise including all of a whole range of possible aspects associated with an individual’s disposition in life.

3 For purposes of this text, “medical student” refers only to a student enrolling into six-year (or equivalent) MBBS (MBChB) or five-year BChD degrees; this naturally excludes pre-med students and students enrolled into nursing or allied health professions degree courses. Dentists are considered medical doctors, for purposes as henceforth presented.

## Appendix 1 – a social blog between nurses

(http://allnurses.com/cna-ma-nursing/hospital-hierarchy-226692.html)

May 27, 2007, by casi

I have an interview with a hospital on Tuesday. I’ve spent the last three years working in assisted living and think that the hospital environment might be an interesting change. My only problem is I know nothing about the hierarchy on a hospital unit and the ranking of positions.

I know that as a CNA I’ll be on the very bottom of the power rankings, and I’m assuming the unit manager I’m interviewing with is at the top for the unit, but who is in between and what are their roles? Who is above and beyond the unit that I may need to know about?

May 28, 2007 by arpeggiated

In my hospital, there’s the CNA (take vitals, clean up patients, organize rooms, collect supplies for procedures/during codes) who takes orders from the nurses. LVNs and RNs are equally respected around here. The LVN works with an RN who signs off on their charting and also hangs blood and does the IV push meds they need.

We have a charge nurse who does the assignments, assigns admissions, schedules meal breaks, enters orders after the secretary leaves at 2330, collects all of our ’junk’ at the end of shift (thermometers, phones, does the narc count, etc) and helps with anything else we need when things go hairy. I LOVE my charge nurse. She is so wonderfully helpful.

We also have a nursing supervisor who assigns beds to admissions, leads codes and the rapid response team (which is when a pt is going bad but hasn’t coded and we want to keep it that way), and takes a basic report from all the nurses to help determine staffing. In addition, the nurses do an ’acuity’ form on the computer measuring things like assesments, treatments, how much time we’ve had to spend with the patient which also helps determine staffing.

Since I live in California, we do have a ratio of only 5 patients per nurse, but if things are crazy, they’ll call more nurses in. No such luck for CNAs! Generally, the max patients our CNAs have is 14 on night shift, which is still a lot when it comes time to take vitals, do I/Os, pass water, etc. Especially if you get lucky and get stuck with a bunch of incontinent patients. During day shift, the DON is there, but I hardly see him (and I like it that way {insert smiley}). Nursing administration is mostly at the other hospital in town, so we don’t see too much of them either.

Other than nursing staff, we have a unit secretary until 2330. She can be your best friend. Our main secretary has been there for DECADES. She knows where everything is, what you need, what the policy is… She can be a lifesaver. She also helps answer call lights. There is also RT (Respiratory) who come and give breathing treatments, suctioning, and just general help when a patient is not breathing right. They tend to be really busy though, because there are only two of them for the whole hospital, and they attend all codes. Then there is the lab and phlebotomists. I usually get asked to come help them hold a confused patient still for blood draws. And there’s housekeeping, who clean rooms and remake the beds after a patient is discharged, bring us supplies (gloves and hand gel), and clean up if a patient has an accident (at least one a week will get in the commode to urinate, and end up peeing all over the floor).

Jun 8, 2007, by Anesthetize

What do you mean by power rankings/hierarchy? Are you getting at your responsibilities relative to others, administrative decisions, giving/taking orders, status, or something else maybe? I don’t see the hospital environment as a power ranking system. Everyone ultimately has a responsibility to the patient. I’d say the patient is the most powerful, and everyone functions within their scope of practice collaboratively or independently to perform certain tasks. Using the term “hierarchies” when talking about job positions usually implies a pecking order and some measure of status, so you’ve got to be careful when and how you use it. A “chain of command,” “order of operations,” or some similar term might be more appropriate. Technically, a “hierarchy” is defined as “the organization of people at different ranks in an administrative body,” and “categorization of a group of people according to ability or status,” among other similar definitions. Technically speaking, hospitals aren’t hierarchies, since there isn’t a ranking system or organization based on status. Hierarchy is too loose of a word to use, much like saying that nurses are middle class citizens, doctors are upper class citizens, CNA’s are lower class citizens, and the administration is the government in hospital land… et cetera et cetera.
